# A novel bicyclic lactone and other polyphenols from the commercially important vegetable *Anthriscus cerefolium*

**DOI:** 10.1038/s41598-022-11923-0

**Published:** 2022-05-12

**Authors:** Rune Slimestad, Bendik Auran Rathe, Reidun Aesoy, Andrea Estefania Carpinteyro Diaz, Lars Herfindal, Torgils Fossen

**Affiliations:** 1 PlantChem AS, Eikenveien 334, 4596 Eiken, Norway; 2 Department of Clinical Science and Centre for Pharmacy, University of Bergen, Jonas Lies vei 87, 5009 Bergen, Norway; 3 Department of Chemistry and Centre for Pharmacy, University of Bergen, Allègaten 41, 5007 Bergen, Norway

**Keywords:** Natural products, Small molecules

## Abstract

Garden chervil, *Anthriscus cerefolium* (L.) Hoffm. is an important herb commonly applied in Norwegian large-scale commercial kitchens. This species is a highly enriched source of phenolics, containing 1260 mg gallic acid equivalents (GAE) 100^–1^ g DM, however, the individual phenolic compounds have been scarcely characterized. Here we report on the qualitative and quantitative content of phenolics in garden chervil. The structure of the main phenolic compound was elucidated to be the previously undescribed compound 1,3-dicaffeoyl-5-malonyl-δ-quinide (**1**) by means of 1D- and 2D NMR and high-resolution mass spectrometry. The known flavones apigenin 7-*O*-β-(2″-apiofuranosylglucopyranoside) (= apiin) (**2**), apigenin 7-(2″-apiosyl-6″-malonylglucoside) (**3**) and luteolin 7-glucoside (**4**) were also identified. Compound **3** is reported for the first time from this plant species. The main phenolic compound, 1,3-dicaffeoyl-5-malonyl-δ-quinide, exhibited moderate cytotoxicity towards acute monocytic leukaemia cells (MOLM-13) and rat kidney epithelial cells (NRK) with EC_50_ between 400 and 600 µM.

## Introduction

Garden chervil or French parsley (*Anthriscus cerefolium* (L.) Hoffm.) is an annual umbelliferous plant with delicate 2- to 3-pinnate leaves and dentated to pinnatisect leaflets. The plant has been utilized as medicinal plant by humankind for millennia and is mentioned in the encyclopedi *Historia naturalis* by Pliny the elder (23–79 ad) as an ingredient in vinegar recommended against hiccup^1^. This species is also mentioned by the seventeenth century Danish pioneer scientist Simon Paulli (1648) who report on its’ use against gallstones^2^. The leaves contain about 0.4% (v/w DM) essential oil, where estragole (= methylchavicol) and 1-allyl-2,4-dimethoxybenzen are the main constituents^3^. The herb has been suggested as a good source for phytosterols and new aroma constituents, which may be useful in human nutrition^4^. The use of Garden chervil as a food in private households seems to be limited as this species is only to a limited extent made available for customers by retailers and supermarkets. However, this species is established as one of the major herbs applied within Norwegian large-scale commercial kitchens^5^, where Garden chervil is used as a cheaper alternative to parsley to give a mild taste of anise, parsley, black pepper and caraway. The knowledge about the phytochemistry and potential health impact of Garden chervil has been studied in current literature and several phenolic constituents of Garden chervil have been characterized. In current literature, several flavones and flavonols have been identified in chervil. The previously reported flavones are all derivatives of apigenin and luteolin, including apiin (apigenin 7-apiosylglucoside)^6,7^, luteolin 7-glucoside, luteolin 7-apiosylglucoside^8^, whereas the identified flavonols are kaempferol, quercetin and isorhamnetin derivatives^7,9^. Several derivatives of quinic acid, in addition to the phenylpropanoids, where the latter compounds occur in the essential oils, have also been reported as phenolic constituents from this species^7,9,10^. These identifications were mainly based on hyphenated mass spectrometry and comparisons with available standards^7^. The observed antimicrobial activity of extracts of chervil may be assigned to the content of relatively volatile compounds including the known antibiotic compound carvacrol^7,11^. Studies of biological activity of pure compounds isolated from chervil are, however, lacking in current literature.

The major objective of this paper is to determine the qualitative and quantitative content of the phenolics of garden chervil by state-of-the-art multidimensional NMR spectroscopic and hyphenated chromatographic methods and to determine the therapeutic potential of the main phenolic constituent towards acute monocytic leukaemia cells.

## Materials and methods

### Reagents and materials

Fresh cut chervil was received from Frøvoll Farm, Randaberg, located in Southwestern Norway (59°0′56.3″ North, 5°37′25.8″ East). Experimental research and field studies on the plants, including the collection of plant material complied with relevant institutional, national, and international guidelines and legislation. Ferric chloride hexahydrate (Fluka), potassium hexacyanoferrate, 2,2-azino-bis (3-ethylbenzothiazoline-6-sulfonic acid) (ABTS), potassium peroxodisulfate, gum Arabic, hydrochloric acid, 85% phosphoric acid, gallic acid, Trolox, potassium phosphate, sodium chloride, chlorogenic acid and formic acid were provided from Merck, Norway. Methanol (Rathburn) and acetonitrile (Rathburn) were provided from Teknolab AS, Norway.

### Sample preparation and analysis

Sample preparations were performed as described by Slimestad et al.^14^. Fresh garden chervil, including leaves and stems, were lyophilized for 72 h using a freeze-dryer (CoolSafe 4 ScanVac, ScanLaf AS, Denmark). Dry matter contents were determined based on the sample weights prior to and after lyophilization. Dried plant material was minced in a bowl chopper and grinded (Bosch KM13, Slovenia). For determination of total phenolic content, radical scavenging capacity and UHPLC analysis, 200 mg of the sample was extracted with 10 mL methanol in a 20 mL test tube at ambient temperature for 48 h in the darkness. Samples were filtered through 0.4 μm syringe filters prior to analysis. Two parallel samples were analysed^14^.

### Fractionation and isolation

Fractionation and isolation of phenolics from garden chervil was based on extracts obtained from 500 g fresh plant material (160 g dry weight). The plant material was minced (about 20 mm), and extraction was performed two times for 24 h in the darkness by using 2 × 500 mL portions of methanol. The extract was filtered (folded filter quality 315, VWR Norway), concentrated to a volume of 100 mL on a rotavapor (Büchi, Switzerland), and partitioned against equal volumes of dichloromethane, in order to remove chlorophylls and lipophilic content. The water phase was further concentrated to a total volume of 50 mL, and the concentrated aqueous extract was applied to a bed of 0.5 kg Amberlite XAD-7 HP (Sigma) in a 5 × 60 cm open-top glass column, rinsed with 2 L distilled water and eluted by use of 2 L MeOH as mobile phase. The XAD-7 purified extract was finally concentrated to a volume of 50 mL.

Further purification was performed by size-exclusion chromatography by using a 5 × 100 cm open-top column filled with 500 g Sephadex LH20 (GE Healthcare, Norway). A step-gradient elution was used with increasing concentrations of methanol in the mobile phase (0, 20, 40, 60 and 80%; 0.1% TFA)^14^. Pure compounds were isolated by preparative HPLC. The HPLC instrument was equipped with a 250 × 20 mm, C18 Ascentis column. Two solvents were used for elution; mobile phase A (water-TFA 99.9:0.1 v/v) and mobile phase B (methanol-TFA 99.9:0.1 v/v). Portions of 200 µL were manually injected into the HPLC column and the collected fractions were transferred to HPLC vials for purity control using analytical HPLC^14^.

### Total phenolic content

Total phenolic content was determined in accordance with the method of Price and Butler with stabilization of the Prussian Blue complex as described by Graham^12,13^ and Slimestad et al.^14^. 100 μL of sample was diluted with 3 mL deionized water and mixed with 1 mL of a 0.1 M ferric chloride in 0.1 M hydrochloric acid solution together with 1 mL of 8 mM potassium ferricyanide solution. The reaction was allowed to run for fifteen minutes at ambient temperature. 5 mL of an acidic gum Arabic solution was added (1 g gum Arabic dissolved in 100 mL hot water. 10 mL of this solution was mixed with 10 mL 85% phosphoric acid and 30 mL water). Absorption at 700 nm was measured by use of an Agilent 8453 spectrophotometer (Agilent Technologies, Matriks, Norway). Samples were measured against a standard curve of gallic acid, and outputs are given as gallic acid equivalents, mg GAE g^−1^^14^.

### Radical scavenging

The TEAC assay (Trolox Equivalent Antiradical Capacity) was carried out following the procedures previously described by Slimestad et al.^14^ and Re et al.^15^. 2,2-azino-bis (3-ethylbenzothiazoline-6-sulfonic acid) (ABTS) was dissolved in water to a 7 mM solution with potassium persulfate to a concentration of 2.45 mM. The solution was kept at ambient temperature for about 16 h. The ABTS^·+^ solution was diluted with PBS (phosphate buffered saline: 100 mM KH_2_PO_4_-buffer, pH 7*.*4 and 150 mM NaCl), to an absorbance of 0*.*70 (± 0*.*02) at 734 nm. Samples were diluted so that, after the introduction of a 10 µL aliquot of each extract into the assay, they produced between 20 and 80% inhibition of the blank absorbance. After addition of 1.0 mL of diluted ABTS^·+^ solution to 10 µL of extracts or trolox standards (final concentration 0–15 µM) in PBS, the absorbance reading was taken at 6 min. Appropriate PBS blanks were run in each assay. The final TEAC decolorization assay values were calculated against a standard curve of 0–10 mg Trolox in 100 mL methanol, and the percentage of inhibition of absorbance at 734 nm were expressed as mg TEAC 100 g^−1^ DW^14,15^.

### U(H)PLC-MS

The qualitative and relative quantitative contents of individual flavonoids and other phenolic compounds was determined by using an Agilent 1290 Infinity II instrument equipped with a 6120 quadrupole mass detector^14^. Separation was achieved with a Zorbax Eclipse XDB-C8 column (2.1 × 100 mm, 1.8 μm, Agilent Technologies). Water with 0.02% HCOOH (solvent A) and acetonitrile (solvent B) were used for gradient elution with the following time program (% B in A): from 0 to 10 (in 1 min), from 10 to 25 (in 25 min), from 25 to 95 (2 min), from 95 to 0 (1 min), and finally isocratic recondition for 1 min. Flow was set to 0.300 mL/min (max back pressure 540 bar), and injections of 5 μL was used. UV-detection was performed at 280, 320 and 360 nm at 4 nm band width. Masses in the range 250–800 Da was detected using a scan time of 500 ms, a fragmentor at 70 V, and detection was in both positive and negative mode^14^. Gas source temperature was set to 350 °C with flow at 10 L min^−1^, nebulizer pressure was 35 psi, whereas capillary voltage was 4 kV^14^.

High-resolution mass spectrometry (LC-HRMS) was used for exact mass determination of isolated compounds. An iClass UPLC (Waters) equipped with a C18 BEH column (1.7 um, 2.1 × 50 mm, Waters) was used for introducing the samples to the mass spectrometer. A gradient of A) 0.2% formic acid and B) acetonitrile was used as follows (% B in A): 1 (isocratic for 0.5 min), from 1 to 90 (in 2 min). The mass spectrometer (timsTOF, Bruker) was used in ESI+-mode with an ionization at 2 kV, and with full scan 100–2000 Da with resolution R = 50,000 (FWHM) at 1000 Da. Exactness at RMS < 1 ppm^14^.

### NMR

NMR samples were prepared by dissolving the isolated compounds in deuterated dimethylsulfoxide (DMSO-D_6_; 99.96 atom % D, Sigma-Aldrich). The 1D ^1^H and the 2D ^1^H–^13^C HMBC, the 2D ^1^H–^13^C HSQC, the 2D ^1^H–^13^C HSQC-TOCSY, the 2D ^1^H–^13^C H2BC, 2D ^1^H–^13^C 1,1 Adequate, the 2D ^1^H–^1^H COSY and 2D ^1^H–^1^H ROESY NMR experiments were obtained at 850 MHz at 298 K on a Bruker 850 MHz instrument equipped with a ^1^H, ^13^C, ^15^N triple resonance cryogenic probe^14^.

### Cytotoxicity assays

The cell lines used to study cytotoxicity were: Molm-13^16^, MV4-11 human monocytic leukemia cells (ATCC, CRL-9591), human acute myeloid leukemia cell line OCI-AML3 (ATCC, ACC-582), NRK rat kidney epithelial cells (ATCC, CRL-6509), and H9c2 cardiomyoblasts (ATCC, CRL-1446). See Bjørnstad et al. 2019 for a description of culture conditions and media for the different cell lines^17^. The cytotoxicity measurements were performed as described earlier^18^. In brief, the compound was dissolved in DMSO, and further diluted in DMSO. Using a pipette robot (Mosquito High Volume, SPT Labtech), 1 L was transferred to 96-well plates to create identical plates for testing on different cells. Cell suspension was then added, and the cells incubated for 72 h before assessment of viability using the WST-1 cell proliferation assay (Roce Applied Sciences). Some experiments were also performed with dilution series performed in culture medium to exclude the effect of DMSO on cell viability. After recording the WST-1 signal, the cells were fixed, DNA stained using Hoechst 33342, and cell death confirmed by microscopic evaluation of nuclear and surface morphology^18^.

## Results and discussion

The dry matter content of chervil, *Anthriscus cerefolium,* was found to be 32.3% which is higher compared to the dry matter content of parsley, *Petroselinum crispum*, at 24.7% provided by the same grower^14^. The total phenolic content was found to be 1260 mg gallic acid equivalents (GAE) 100 g^−1^, quite similar to that of parsley (1270 mg GAE 100 g^−1^)^14^. The antiradical potential was found to be 50 ± 5 mg TEAC 100 g^−1^, similar to the level detected in parsley, 54 mg TEAC 100 g^−1^^14^ and comparable to previous studies of chervil^11^.

**Figure 1:**
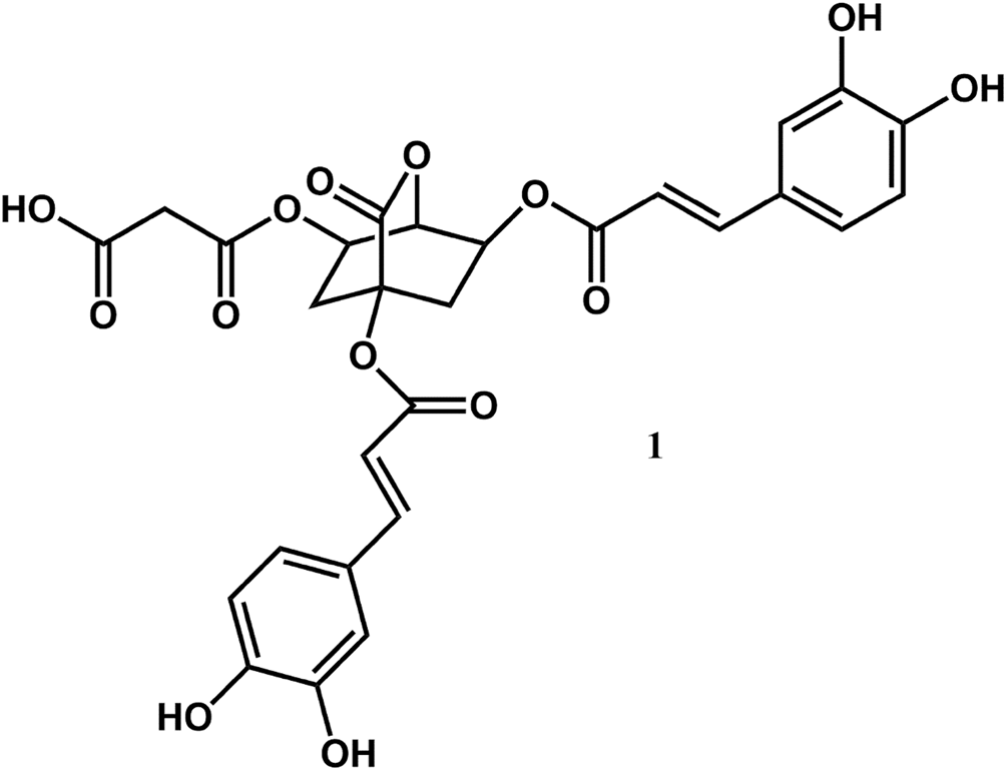
Structure of the previously undescribed compound 1,3-dicaffeoyl-5-malonyl-δ-quinide (**1**) isolated from *Anthriscus cerefolium*. Structure was drawn in ChemDraw Professional 18.0. https://www.alfasoft.com/no/produkter/lab/chemdraw.html.

From a methanol extract of fresh garden chervil (*Anthriscus cerefolium* L.) four phenolic compounds were detected by means of UHPLC-DAD-MS analysis (Fig. 1, Table 1). Preparative isolation of the phenolics was achieved by repetitive size-exclusion chromatography (Sephadex LH-20) and preparative HPLC. In total five compounds were isolated. Compounds **2**, **3** and **4** were identified as apigenin 7-*O*-β-(2″-apiofuranosylglucopyranoside) (**2**, apiin), luteolin 7-*O*-β-glucopyranoside (**3**), and apigenin 7-*O*-β-(2″-apiofuranosyl-6″-malonylglucopyranoside) (**4**) by a combination of several 1D and 2D NMR experiments (Tables S1, S2) and high-resolution mass spectrometry (Table 1). Apiin and luteolin 7-*O*-β-glucopyranoside have previously been identified in chervil^6,7^, while apigenin 7-*O*-β-(2″-apiofuranosyl-6″-malonylglucopyranoside) is identified in garden chervil for the first time.

**Table 1:** UHPLC-MS and HRMS data of main phenolics in methanolic extracts of chervil, *Anthriscus cerefolium*. Characteristic ions (*m/z*) from mass detection is listed with the pseudomolecular ions ([M + H]^+^ or [M-H]^−^) followed by fragments ions included those of the aglycone moieties.

*t*_R_ (min)	λ_max_ (nm)	Pos./neg. ions	HRMS [M + H]^+^	Calc. formula	Compounds
7.82	266, 336	565, 271 /563	565.1547	C_26_H_29_O_14_	Apigenin 7-apiosylglucoside (apiin) (**2**)
8.31	300sh, 330	585, 423 /601	585.1234	C_28_H_25_O_14_	1,3-Dicaffeoyl-5-malonyl-δ-quinide (**1**)
ND	ND	ND	449.1076	C_2_^1^H_21_O_11_	Luteolin 7-glucoside (**4**)
9.42	266, 336	651, 271 /649	651.1544	C_29_H_31_O_17_	Apigenin 7-malonylapiosylglucoside (**3**)

*Nd* not determined.

The central bicyclic δ-quinide ring system of **1** was assigned by the ^1^H/^13^C crosspeaks at δ 2.44/69.9 (H2A/C3), δ 1.96/69.9 (H2B/C3), δ 5.20/35.7 (H3/C2), δ 5.20/68.8 (H3/C4), δ 3.87/69.9 (H4/C3), δ 3.87/72.0 (H4/C5), δ 5.32/68.8 (H5/C4), δ 5.32/31.8 (H5/C6) and δ 2.41/72.0 (H6/C5) observed in the 2D ^1^H–^13^C H2BC spectrum, the ^1^H/^1^H crosspeaks at δ 2.44/5.20 (H2A/H3), δ 1.96/5.20 (H2B/H3), δ 2.44/1.96 (H2A/H2B), δ 5.20/3.87 (H3/H4), δ 3.87/5.32 (H4/H5) and δ 5.32/2.41 (H5/H6) observed in the 2D ^1^H–^1^H COSY spectrum. The substituents of the central bicyclic δ-quinide ring system of **1** were identified as two caffeoyl units and a malonyl unit (Table 2). The ^1^H/^13^C crosspeaks at δ 6.28/78.8 (H8′/C1), δ 6.23/69.9 (H8″/C3) and δ 5.20/166.2 (H3/C9″) confirmed that the caffeoyl units were attached to the δ-quinide ring system at position 1 and 3, respectively. A crosspeak at δ 5.32/166.7 (H5/C1‴) confirmed the linkage between the malonyl substituent and the δ-quinide ring system at position 5. Thus, **1** was identified as the previously undescribed natural product 1,3-dicaffeoyl-5-malonyl-δ-quinide. A molecular ion at *m/z* 585.1234 corresponding to C_28_H_25_O_14_ (calculated 585.1245, δ = − 1.9 ppm) observed in the high-resolution mass spectrum of **1** confirmed this identity.

**Table 2:** ^1^H and ^13^C NMR chemical shift values (ppm) and coupling constants (Hz) of 1,3-dicaffeoyl-5-malonyl-δ-quinide (**1**) isolated from *Anthriscus cerefolium* recorded in DMSO-D_6_ at 298 K.

	δ ^1^H	δ ^13^C
1		78.82
2A	2.44 dd 4.4, 14.0	35.7
2B	1.96 m	
3	5.20 dt 4.4, 9.0	69.86
4	3.87 dd 3.7, 8.8	68.8
5	5.32 dd, 4.4, 8.1	71.98
6	2.41 m	31.83
7		172.08
**1-*O*-*Z*-caffeoyl**		
1′		125.59
2′	7.08 d 2.1	115.25
3′		145.80
4′		148.83
5′	6.78 d 8.2	116.02
6′	7.02 dd 2.1, 8.2	121.67
7′	7.49 d 15.7	146.26
8′	6.28 d 15.7	113.89
9′		165.43
3′-OH	9.19 s	
4′-OH	9.67 s	
**3-*O*-*Z*-caffeoyl**		
1″		125.70
2″	7.06 d 2.1	114.98
3″		145.80
4″		148.69
5″	6.77 d 8.2	115.99
6″	6.99 dd 2.1, 8.2	121.67
7″	7.49 d 15.7	145.66
8″	6.23 d 15.7	114.13
9″		166.17
3″-OH	9.21 s	
4″-OH	9.63 s	
**5-*O*-malonyl**		
1‴		166.65
2A‴	3.31 d 15.9	41.74
2B‴	3.22 d 15.9	
3‴		167.94
3‴-OH	12.5 s (br) (very broad)	

A related compound with similar central bicyclic δ-quinide ring system to that of **1**, namely 3,5-O-dicaffeoyl-epi-δ-quinide, has previously been reported to occur in processed coffee beans, where the identification was solely based on mass spectrometry^19^. Recently, Stojkovic et al. reported the presence of several derivatives of quinic acid acylated with caffeoyl and malonyl moieties, which were identified by hyphenated high resolution mass spectrometry (UHPLC-MS)^7^, however, none of these compounds were lactones.

**Figure 2:**
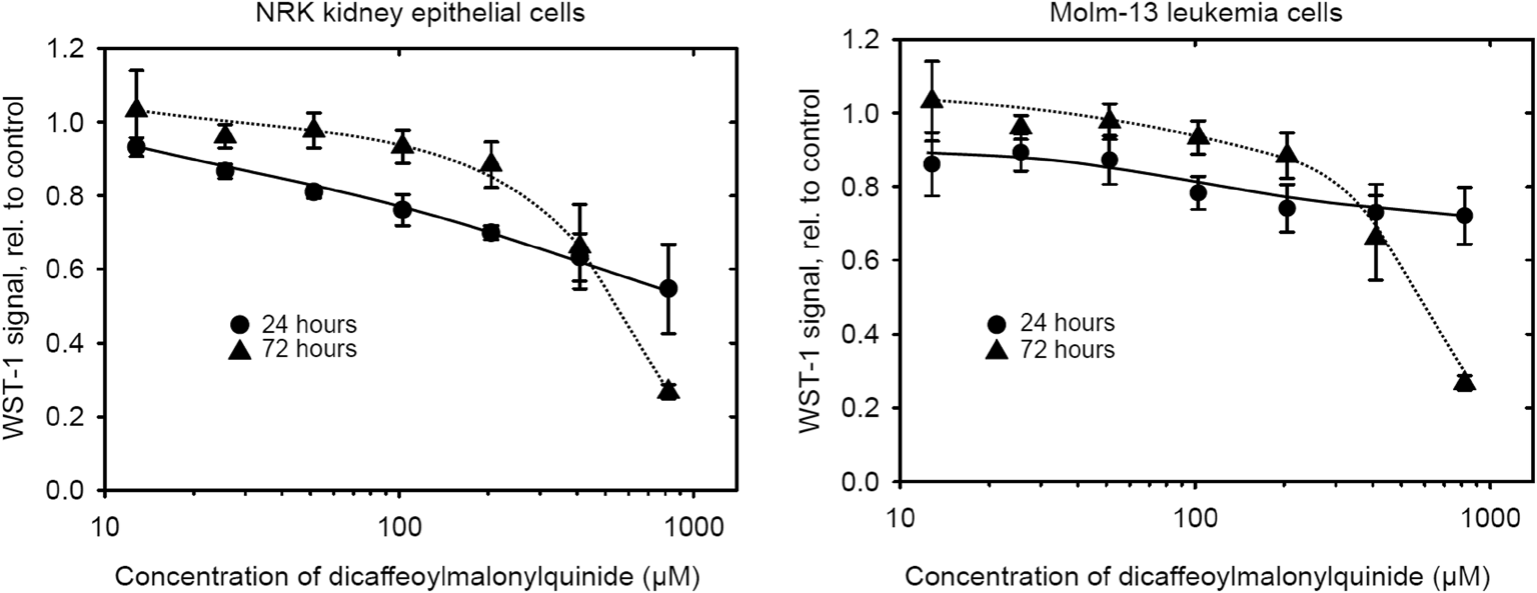
Cytotoxicity of 1,3-dicaffeoyl-5-malonyl-δ-quinide (**1**) towards NRK kidney epithelial cells and Molm-13 leukemia cells. The figure was created with SigmaPlot 14 (Systat Software, Inc., San Jose, CA, USA). https://systatsoftware.com/sigmaplot/.

Previously, extracts of chervil have exhibited weak to moderate cytotoxic activity towards cancer cell lines including gliblastoma cells, where EC_50_ of 765 µg/mL was observed^7,20^, however, the cytotoxicity of pure compounds isolated from chervil has not been reported in current literature. The cytotoxicity of the main phenolic compound 1,3-dicaffeoyl-5-malonyl-δ-quinide (**1**) towards MOLM13, OCI-AML3 and MV4-11 acute leukaemia cells and towards normal cell lines (NRK cells and H9C2) (Fig. 2 shows results on MOLM13 and NRK cells after 24 and 72 h incubation). 1,3-dicaffeoyl-5-malonyl-δ-quinide (**1**) exhibited moderate toxicity towards all cell lines tested with EC_50_ values above 1000 µM at 24 h incubation, and between 400 and 600 µM at 72 h incubation (Fig. 2 and data not shown). The compound did not show selective cytotoxicity towards any of the leukemia cell lines compared to the non-cancerous cell lines.

## Conclusions

Chervil (*Anthriscus cerefolium* (L.) Hoffm) is a very rich source of phenolic compounds, 1260 mg GAE 100^–1^ g DM, and its methanolic extract has a high antiradical capacity. Interestingly, though the two species contain the same main flavones (apiin and apigenin 7-(2″-apiosyl-6″-malonylglucoside)), the major phenolic constituent of chervil is a bicyclic lactone; 1,3-dicaffeoyl-5-malonyl-δ-quinide (**1**). This compound is reported for the first time. 1,3-dicaffeoyl-5-malonyl-δ-quinide exhibited moderate cytotoxic activity towards human leukaemia and normal cell lines.

## Supplementary Information

Supplementary Tables.
