# Supplementary material for: A novel bicyclic lactone and other polyphenols from the commercially important vegetable Anthriscus cerefolium
Source: Sci Rep. 2022 May 12;12:7805. doi: 10.1038/s41598-022-11923-0 (PMC9098402; doi:10.1038/s41598-022-11923-0)
Supplement: Supplementary file 1 — Supplementary Tables. [file 41598_2022_11923_MOESM1_ESM.pdf]

Supplementary material

A novel bicyclic lactone and other polyphenols from *Anthriscus cerefolium* (L.) Hoffm., an important vegetable in Norwegian large-scale commercial kitchens

Rune Slimestad, Bendik Auran Rathe, Reidun Æsøy, Andrea Estefania Carpinteyro Diaz, Lars Herfindal and Torgils Fossen

Table S1. ^{1}H NMR chemical shift values (δ ^{1}H, ppm) and coupling constants (Hz) of apigenin 7-(2’’-apiosylglucoside) (**2**, apiin), apigenin 7-(2’’-apiosyl-6’’-malonylglucoside) (**3**) and luteolin 7-*O*-β-glucopyranoside (**4**) isolated from *Anthriscus cerefolium* recorded in DMSO-D_6 at 298K.

	**2**	**3**	**4**
3	6.83 s	6.86 s	6.74 s
6	6.42 d 2.2	6.41 d 2.2	6.43 d 2.2
8	6.80 d 2.2	6.77 d 2.2	6.78 d 2.2
2’	7.93 ‘d’ 8.7	7.95 ‘d’ 8.9	7.42 d 2.4
3’	6.93 ‘d’ 8.7	6.93 ‘d’ 8.9	
5’	6.93 ‘d’ 8.7	6.93 ‘d’ 8.9	6.90 d 8.3
6’	7.93 ‘d’ 8.7	7.95 ‘d’ 8.9	7.44 dd 2.4, 8.3
5-OH	12.95 s		
5-OH		12.97 s	
4’-OH	10.39	10.42 s	
7-*O*-*β*-glucopyranoside			
1’’’	5.16 d 7.7	5.20 d 7.8	5.07 d 7.7
2’’’	3.53 dd, 7.7, 9.1	3.54 m	3.25 dd 7.7, 9.0
3’’’	3.49 t 9.1	3.49 m	3.29 t 9.0
4’’’	3.20 t 8.9	3.21 m	3.17 dd 9.0, 9.5
5’’’	3.48 m	3.78 ddd 2.1, 7.0, 9.6	3.43 ddd 9.5, 5.8, 2.1
6A’’’	3.72	4.39 dd 2.1, 12.0	3.70 dd 2.1, 11.9
6B’’’	3.48 m	4.11 dd 7.0, 12.0	3.47 dd 5.8, 11.9

2’’-*O*-apiofuranosyl		
1’’’	5.35 d 1.2	5.34 d 1.4
2’’’	3.76 d 1.2	3.74 d 1.4
4A’’’	3.92 d 9.4	3.91 d 9.5
4B’’’	3.66 d 9.4	3.66 d 9.5
5A’’’	3.33 d 11.2	3.31 m
5B’’’	3.30 d 11.2	3.28 m
6’’-*O*-malonyl		
2A’’’		3.40 d 15.7
2B’’’		3.36 d 15.1

Table S2. ^{13}C NMR chemical shift values (δ ^{13}C, ppm) of apigenin 7-(2''-apiosylglucoside) (**2**, apiin) apigenin 7-(2''-apiosyl-6''-malonylglucoside) (**3**) and luteolin 7-*O*-β-glucopyranoside (**4**) isolated from *Anthriscus cerefolium* recorded in DMSO-D_6 at 298K.

	**2**	**3**	**4**
2	164.41	164.43	164.8
3	103.23	103.18	103.4
4	182.13	182.10	182.1
5	161.29	161.21	161.7
6	99.48	99.45	99.7
7	162.83	162.49	163.2
8	94.94	94.76	94.9
9	157.06	157.02	157.2
10	105.52	105.56	105.5
1'	121.17	121.08	121.6
2'	128.75	128.68	113.8
3'	116.14	116.08	146.0
4'	161.50	161.45	150.1
5'	116.14	116.08	116.8
6'	128.75	128.68	119.4
7-*O*-*β*-glucopyranoside			
1'''	98.27	97.90	100.1
2'''	75.89	75.56	73.3
3'''	76.94	76.53	76.6
4'''	69.94	69.90	69.8
5'''	77.17	73.72	77.4
6'''	60.67	64.06	60.8
2''-*O*-apiofuranosyl			
1'''	108.88	108.83	
2'''	76.22	76.15	
3'''	79.44	79.38	
4A'''	74.14	74.07	

5A’’’	64.36	64.36
6’’-*O*-malonyl		
1’’’		166.87
2’’’		41.41
3’’’		167.85
